# Nuclear position relative to the Golgi body and nuclear orientation are differentially responsive indicators of cell polarized motility

**DOI:** 10.1371/journal.pone.0211408

**Published:** 2019-02-13

**Authors:** Megan E. Brasch, Giuseppe Passucci, Anushree C. Gulvady, Christopher E. Turner, M. Lisa Manning, James H. Henderson

**Affiliations:** 1 Department of Biomedical and Chemical Engineering, Syracuse University, Syracuse, NY, United States of America; 2 Syracuse Biomaterials Institute, Syracuse University, Syracuse, NY, United States of America; 3 Department of Physics, Syracuse University, Syracuse, NY, United States of America; 4 Department of Cell and Developmental Biology, SUNY Upstate Medical University, Syracuse, NY, United States of America; University of California San Diego, UNITED STATES

## Abstract

Cell motility is critical to biological processes from wound healing to cancer metastasis to embryonic development. The involvement of organelles in cell motility is well established, but the role of organelle positional reorganization in cell motility remains poorly understood. Here we present an automated image analysis technique for tracking the shape and motion of Golgi bodies and cell nuclei. We quantify the relationship between nuclear orientation and the orientation of the Golgi body relative to the nucleus before, during, and after exposure of mouse fibroblasts to a controlled change in cell substrate topography, from flat to wrinkles, designed to trigger polarized motility. We find that the cells alter their mean nuclei orientation, in terms of the nuclear major axis, to increasingly align with the wrinkle direction once the wrinkles form on the substrate surface. This change in alignment occurs within 8 hours of completion of the topographical transition. In contrast, the position of the Golgi body relative to the nucleus remains aligned with the pre-programmed wrinkle direction, regardless of whether it has been fully established. These findings indicate that intracellular positioning of the Golgi body precedes nuclear reorientation during mouse fibroblast directed migration on patterned substrates. We further show that both processes are Rho-associated kinase (ROCK) mediated as they are abolished by pharmacologic ROCK inhibition whereas mouse fibroblast motility is unaffected. The automated image analysis technique introduced could be broadly employed in the study of polarization and other cellular processes in diverse cell types and micro-environments. In addition, having found that the nuclei Golgi vector may be a more sensitive indicator of substrate features than the nuclei orientation, we anticipate the nuclei Golgi vector to be a useful metric for researchers studying the dynamics of cell polarity in response to different micro-environments.

## Introduction

The organization and reorganization of intracellular structures and organelles is key to the complex biological processes of both cell motility and collective cell behaviors at the tissue scale. For example, fixed slide images of stained nuclei and microtubule-organizing centers (MTOCs) have implicated these organelles in fibroblast wound-edge polarization and cell-cell contact polarity [1]. Indeed, during the process of polarization and directed motility, both the MTOC and Golgi become positioned towards the wound edge while the nucleus becomes positioned away from the leading edge, with coordination of these events dependent on the small RhoGTPase Cdc42 [1–4]. The repositioning of the Golgi apparatus contributes to polarized cell migration by facilitating the efficient transfer of Golgi-derived vesicles, via microtubules, to the cells leading edge [5, 6]. These vesicles provide the membrane and associated proteins necessary for directed lamellipodial protrusion [7]. Importantly, the timing of Golgi repositioning in relation to changes in overall cell morphology and intracellular signaling remain poorly understood.

Despite the recognized involvement of organelles in cell motility, the role of organelle positional reorganization in cell motility is not entirely clear, in part due to limitations of existing experimental approaches. In particular, the existence of simultaneous biochemical and biomechanical signaling has complicated *in vivo* efforts to understand the forces regulating intracellular reorganization, individual cell motility, and collective cell behaviors [8]. This coupling can be especially challenging to unravel for processes in which extracellular signals evolve over long timescales (e.g., hours to days). The spatial organization and reorganization of intracellular structures and organelles that gives rise to polarized motility in structured environments is such a process.

To better understand the complex relationship between organelles and cell motility, we recently developed software to track thousands of cell nuclei over long time periods (24 h) [9] and applied it to the study of cells on programmable shape memory polymer (SMP) substrates. SMP substrates and scaffolds have emerged as experimental platforms that can help isolate the relationship between biomechanical changes in the microenvironment and cellular responses (e.g., cell alignment or motility). SMPs can be fabricated in an initial shape and then temporarily “fixed” in a secondary shape that can be triggered via stimuli such as heat, hydration, or electrical current to change shape back to the initial shape [10], thereby dynamically altering, with a controlled timescale, the extracellular environment of cells cultured thereon. We [11–14] and others [15–20] have demonstrated that SMP-actuated changes induce cytoskeletal and nuclear reorganization of cells to realign to changes in 2D topographies and 3D structures.

We have previously applied the computational cell-tracking system to characterize on-command on/off switching of polarized motility responses to changes in SMP scaffold architecture. The biomechanical signaling provided by the SMP allowed study of polarization and motility, as might occur during a process such as wound-edge polarization or cell-cell contact polarity, without the confounding simultaneous biochemical signaling that generally exists *in vivo*. That work demonstrated a clear correlation between nuclear alignment, cell body alignment, and directional motility in dynamically changing environments [21]. While the work investigated the cellular response before and after a biomechanical change in a 3D environment, it did not explore how intracellular reorganization or cell motility responses varied *during* the dynamic change in the extracellular environment. Additionally, other organelles likely to provide critical insight into polarized motility, particularly the Golgi body, were not studied. Monitoring the position of both the nucleus and other organelles that rearrange during directed migration may provide greatly improved understanding and experimental metrics for determining and quantifying cell orientation.

To improve understanding of how organization and reorganization of intracellular organelles regulate cell motility, the goal of the present study was to examine the temporal evolution of mouse fibroblast polarization through monitoring the position and shape of the nucleus and Golgi body in response to changes in substrate topography. To achieve this goal, a novel tracking process was developed to identify and track Golgi bodies and link them positionally with their respective cell nuclei. This process was combined with our established approach for tracking cell nuclei [9] to study mouse fibroblast motility on SMP substrates as they transitioned from a flat surface to an aligned nanowrinkled topography.

## Materials and methods

### Substrate preparation

Poly(tert-butyl acrylate-co-butyl acrylate) (tBA:BA) SMP films were prepared as previously reported [12]. 95:5 wt% tBA:BA films were fabricated using 5 wt% tetraethylene glycol dimethacrylate as crosslinker and 0.06 wt% 2,2-dimethoxy-2-phenylacetophenone as a photoinitiator. Samples were cured for 30 mins under UV light, followed by extraction in a 1:1 solution of methanol and distilled water overnight. Samples were then dried for at least 2 days in a 40°C vacuum oven prior to sample processing. SMP films were processed in one of three ways: 1) as static, flat controls (hereafter referred to as non-wrinkled, NW), 2) as static, wrinkled controls (hereafter referred to as wrinkled, W), or 3) as the active wrinkling experimental group (hereafter referred to as active, A). Static non-wrinkled samples were cut into 6x6 mm squares and heated to 80°C on a hotplate to ensure no surface flaws were present. Static wrinkled and active wrinkling films were strained 7% in tension in an 80°C isothermal oven for 10 minutes and subsequently cooled at -4°C for 5 mins to fix in the strain. Wrinkled and active groups were cut into 6x6 mm squares using a hammer and razorblade in order to avoid premature recovery of the samples due to the heating that can accompany alternative methods of cutting. All three sample groups were then sputter coated with gold for a total of 100 secs, resulting in an approximately 33 nm thick coating on the material surface. Wrinkled samples were recovered for 2 hrs at 60°C in an isothermal oven, resulting in a nanotopographic pattern with features on the order of 400 nm in amplitude and 1–5 um in wavelength [12]. All sample groups were then UV sterilized for one hour on each side in a biological safety cabinet (ThermoFisher, 1300 Series A2) for subsequent cell culture.

### Fibroblast cell culture, golgi infection, and time-lapse imaging

C3H10T1/2 mouse fibroblast cells (ATCC) were cultured in Basal Medium Eagle (BME) complete growth medium supplemented with 10% fetal bovine serum (FBS) (v/v), 1% penicillin/streptomycin (v/v), and 1% GlutaMax (v/v). Cells were expanded in a 37°C humidified incubator with regulated 5% CO_2_ and passaged at 80% confluence using 0.25% Trypsin EDTA. For time-lapse experiments, cells were restricted to passage numbers 12–18.

To enable visualization and tracking of the Golgi, cells were infected with a biological marker of the Golgi cisternae. Briefly, cells were passaged using 0.25% Trypsin EDTA and plated at 50,000 cells/well in 1 mL of media (per well) in a 6-well plate. Cells were then infected with an average of ~30 particles per cell of CellLight Golgi-RFP, BacMam 2.0 (ThermoFisher Scientific). 1 μL of Bacmam Enhancer (ThermoFisher Scientific) was added per well to improve infection efficiency to ~70%. Infected cells were then cultured for 24 hrs in a 37°C humidified incubator with 5% CO_2_ to ensure cell uptake of the viral particles.

Prior to cell seeding, SMP samples were soaked in room temperature BME medium for 6 hrs to promote FBS protein adsorption to the material surface. RFP infected cells were then passaged using 0.25% Trypsin EDTA warmed to 30°C. Each sample was transferred into an individual well in a 48-well plate and cells were solution seeded (500 μL/well) at a density of 4000 cells/cm^2^. Cell samples were then incubated at 30°C for 16 hrs to establish a relative equilibrium of cell motility prior to time-lapse image set-up. Hoechst dye was added to fresh BME complete medium at a concentration of 0.01 ug/ml as previously described [9].

To investigate the underlying signaling mechanisms, we studied the extent to which Rho signaling affects the response of cells to static and dynamic topographies by inhibiting Rho kinase (ROCK) activity through the addition of 10 μM Y-27632 ROCK inhibitor (Calbiochem) [22].

To stain the nuclei and image the cells, 800 uL of the staining or staining/ROCK solution were added to each well of a 4-well LabTek borosilicate chamber slide (Fisher Scientific). Static wrinkled, static non-wrinkled, and active wrinkling samples were transferred into the chamber slide and incubated at 30°C for 1 hr. Samples were then inverted and weighed down with sterilized glass slide inserts, cut to fit into the chamber wells. The chamber slide was then transferred to a live-cell stage incubator (INC-2000, 20/20 Technology, Inc.) and cells were imaged using a Leica DMI 6000B inverted fluorescence microscope. The live-cell stage incubator was equilibrated at 30°C with constant 5% CO_2_. One image per position of interest was captured every five minutes in each of phase, A4 (excitation/emission peak of 360/470 nm), and N3 (excitation/emission peak of 546/600 nm) using 50 ms, 100 ms, and 50 ms exposure times respectively on an Andor Luca R camera with a 10x/0.63 NA objective. Samples were imaged in succession for 4 hrs at 30°C, followed by 20 hrs at 37°C. Video was captured at a minimum of three positions on each substrate, replicated at least three times for each substrate type. The shape-memory polymer properties of the specific synthetic batches chosen for the active group were such that active group substrates showed very slow recovery during the 4 hrs at 30°C, resulting in slow appearance of substrate anisotropy and topography during that period, followed by more rapid and pronounced topographic shape change upon heating to 37°C, with completion of formation of the nano-wrinkled topography during the subsequent isothermal culture.

### Dual nuclear and golgi tracking

**Fig 1:**
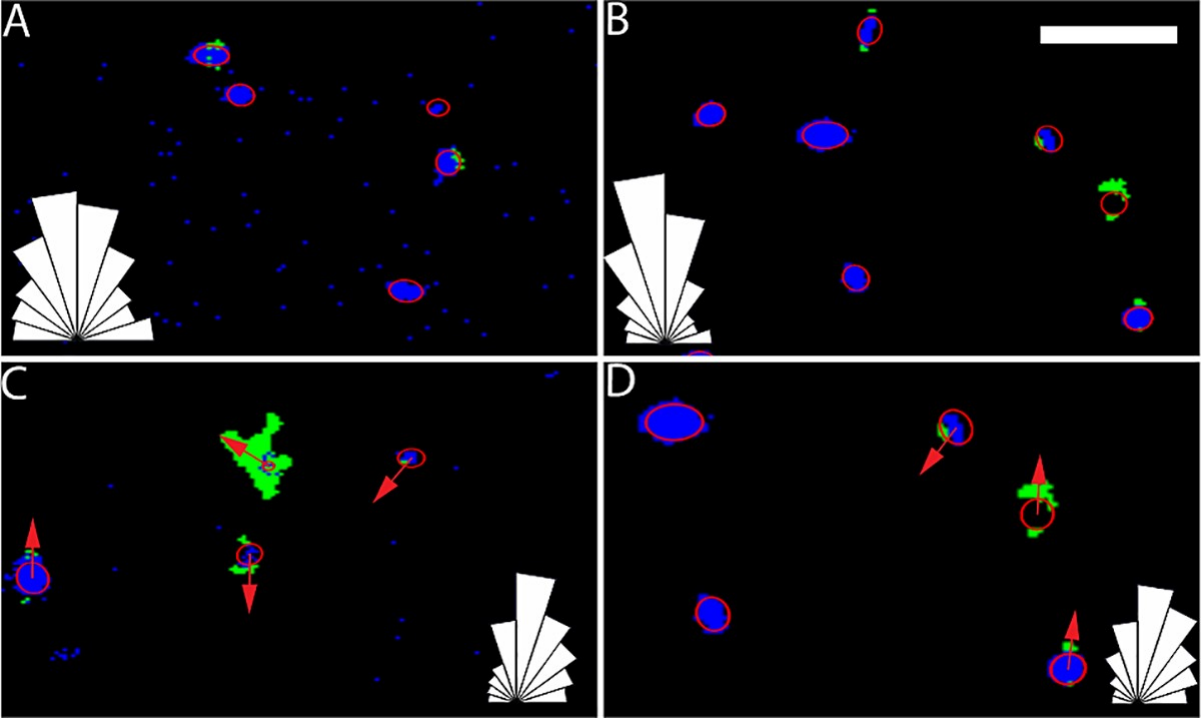
Representative example of the process for dual nuclear and Golgi tracking. Image tracking. Images of nuclei (blue) and Golgi bodies (green) are shown before (A,C) and after (B,D) activation, where the wrinkle direction is vertical in all images shown. Nuclei were fit with ellipses (shown in red) before (A) and after (B) wrinkling, with orientation defined as the ellipse major axis. In addition, we identified both the nucleus and Golgi body to determine cell orientation before (C) and after (D) wrinkling, indicated by red arrows between a Golgi body and nucleus in the same cell. Polar histograms in the bottom right show a similar mean and truncated standard deviation. Scale bar is 100 μm.

To generate the polarization data (Fig 1), live-cell time-lapse motility videos were characterized using a combination approach of ACT*IV*E nuclear tracking [9] and the new Golgi body tracking code. This Golgi body tracking software (see Supporting Information for details) was used to correlate Golgi body motion to nuclear directional behaviors in order to track cell polarization over time as the cells were exposed to both the initial gradual change in topography and the subsequent more rapid change. We identified cell nuclei before and after the wrinkling activation (Fig 1A and 1B) and also tracked Golgi bodies to construct another metric for orientation, the vector that connects the center of mass of the nucleus to the Golgi (Fig 1C and 1D), with corresponding polar histograms used to report the aggregate data.

To track and quantify orientation for a large number of nuclei across many samples, we used ACT*IV*E, an accurate, robust cell tracking algorithm developed for analyzing migratory behaviors of adherent cells stained, infected, or transfected with a nuclear marker that has successfully been used to analyze C3H10T1/2 mouse fibroblast [9, 21], HT-1080 human fibrosarcoma [21], and Escherichia coli [23, 24] migratory behaviors. The accuracy of ACT*IV*E when compared to manual tracking has been reported previously [9]. ACT*IV*E was applied here as previously described [9].

Although ACT*IV*E was very successful at tracking cell nuclei, it relied heavily on their ellipsoidal shape. In contrast, the Golgi body varies substantially in density and can have a very irregular shape. To overcome this challenge, we used a hard-cutoff threshold to remove background noise and the *clusterData* MATLAB function [25] to roughly group pixels. We developed an additional algorithm to combine (associate) cluster fragments into the correct parent Golgi bodies, using a single input “distance” parameter that identifies a rough estimate of the average expected distance between Golgi bodies, determined by the seeding density, transfection efficiency, and micron-to-pixel ratio. Golgi bodies across frames were linked using an established method [26] and successfully tracked nuclei and Golgi bodies were then linked together to create a master identification list.

### Cell speed

Cell speed was calculated from changes in nuclei center-of-mass frame-to-frame. This positional data was obtained from the ellipses fit by the ACT*IV*E tracking package. The ACT*IV*E software contains a "memory" parameter, which represents the number of frames the linking code will allow between positions for a nucleus to retain the same identification number. ACT*IV*E utilizes the feature controlled by this parameter, which is standard in many particle tracking algorithms [26, 27], to more accurately track cells over time. More specifically, since cells (and organelles in this particular case) display variability in fluorescent signal intensity over time, every cell or organelle will not necessarily be identified in every video frame. Similarly, as cells interact with one another, the algorithm may identify two interacting cells as a single cell. The memory parameter allows the ACT*IV*E system to retain information on cells that may be absent for one or more frames. This feature was optimized for the original ACT*IV*E tracking study via a comparison to manual tracking data [9] to allow for cells to “disappear” for a maximum of 10 frames prior to being assigned a new ID for tracking upon reappearing. Since the imaging conditions for fluorescently labeled nuclei were very similar between this study and reference [9], we retained the memory parameter of 10 for this study. Visual inspection comparing image stacks and tracked trajectories confirmed that this parameter choice performed well for the data sets in this manuscript. This, in combination with a cost function, allows cells to accurately be tracked over long timescales (≥24 h) and in dense environments (1000+ cells per field of view). Due to this feature, gaps in positional data as a function of time may occur. Since the speed represents a change in position, we filled these gaps by assuming that, while a nucleus was missing, it was travelling in a straight line between the last known position before it disappeared and the first position after it reappeared. Given the small number of frames for potential gaps, the distance interpolated was negligible and allowed us to smoothly calculate the speed with these filled-in trajectories. We then examined the absolute value of change in the x-direction to calculate the x-component of the velocity and performed an analogous process for the y-direction. The x-axis was always rotated to correlate with the wrinkle direction, where one existed. We combined these results to calculate the speed at which the nuclei centers-of-mass were moving frame to frame and to ultimately plot the mean squared displacement of cells over time.

### Nuclear and nuclear-golgi truncated standard deviation

A focus and main contribution of the present work was in improving both the tools available to study and the understanding of cell polarization responses by developing an approach for analyzing and quantifying nuclei orientation and the nuclear-Golgi polarization vector formed by pairing the nucleus and Golgi body orientation from the same cell over time. As previously noted, the Golgi body orients toward the leading edge while the nucleus orients towards the rear of the cell. Therefore, we can use the nuclei Golgi vector as a metric for internal cell polarization that can be compared to nuclei orientation. We treated all of the directions as apolar (so that angles of 0 and 180 degrees are equivalent), as we were primarily concerned about whether the cells were aligning to the surface topography. To calculate the nuclear and nuclear-Golgi orientation, we used the truncated standard deviation [28]. The truncated standard deviation was chosen for this analysis, because, unlike the standard deviation, the truncated standard deviation can quantify angular variability in both random and highly aligned systems, thereby providing a metric that facilitates direct comparison across systems of varying alignment. All angles were first wrapped between [1°, 180°]. Next the standard deviation, σ, of this distribution was calculated. From this, the truncated standard deviation was calculated:

$$\begin{array}{c} \sigma_{t}=\frac{52}{\left(1+543*\sigma^{-1.96}\right)} \end{array}$$ (1)

A uniform random distribution of σ_t_ would generate an angular spread of 52°, while perfect alignment would result in an angular spread of 0°. Therefore, smaller values indicate more highly aligned cells. After calculating the angular spread, the distribution was shifted by one degree and re-wrapped from [1°, 180°]. The mean of the distribution was then calculated and the process was repeated until the reference angle reached 180°. The mean distribution with the smallest truncated standard deviation was identified as the mean orientation for the system.

### Statistics

Kruskal-Wallis nonparametric testing with post-hoc Bonferroni corrections was used for statistical comparisons. A nonparametric design was chosen due to the potentially non-normal distribution of cell data. Significance was determined at 95% and 99% p-values.

### Radius of gyration

The radius of gyration, R_G_, quantifies the amount of space that a cell explores over time [29]. We constructed the full radius of gyration tensor and then compared the diagonal elements R_xx_ and R_yy_, which quantify cell exploration along the x and y directions, respectively. By averaging over all cell trajectories and taking a ratio of these two quantities, we generated a metric for the anisotropy of cell trajectories. Values of R_xx_/R_yy_ below unity indicate that cells have traveled farther perpendicular to the x-axis, while values above unity indicate preferential travel along the x-axis direction.

### Velocity auto-correlation function (VACF)

We calculate the standard directional velocity auto-correlation function:

$$C_{vv}=<\vec{v}(t_{0})\vec{v}(t_{0}+dt)>=\frac{\vec{v}(t_{0})}{\left|\vec{v}(t_{0})\right|}\frac{\vec{v}(t_{0}+dt)}{\left|\vec{v}(t_{0}+dt)\right|},$$

where the velocities, $\vec{v}(t)$, are the instantaneous displacements between two sequential frames and the brackets indicate averaging over the ensemble and initial times *t*_0_.

## Results and discussion

### Shape memory polymer characterization

**Fig 2:**
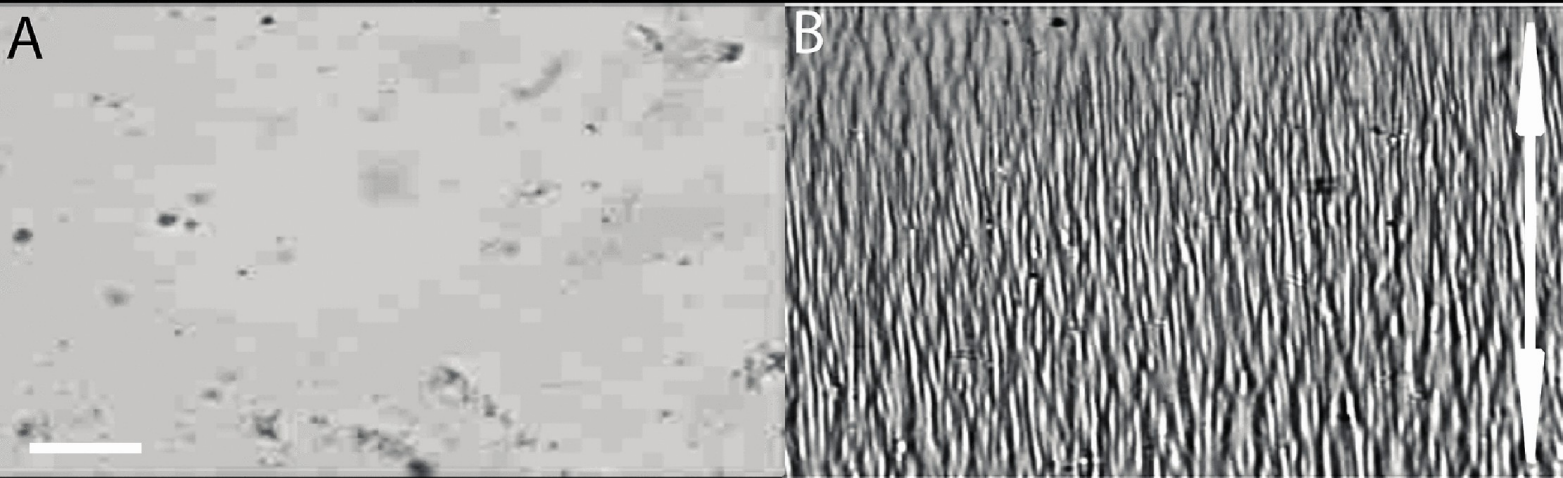
Topographic change of the shape memory polymer substrates. Optical micrographs of an active wrinkling shape memory polymer substrate (A) before and (B) after completion of formation of the nano-wrinkled topography employed to examine the temporal evolution of mouse fibroblast polarization. The direction of programmed tensile strain in (A) is horizontal, and, thus, the direction of wrinkle formation in (B) is vertical, as indicated by the double-headed arrow. Scale bar is 50 μm.

Optical micrographs of an active SMP substrate in its fixed, flat state pre-activation (Fig 2A) and subsequently in its recovered, wrinkled topography post-activation (Fig 2B) confirmed the intended change in topography in the active SMP substrate system.

### Cell motility is directed along wrinkles in active and static wrinkled substrates

**Fig 3:**
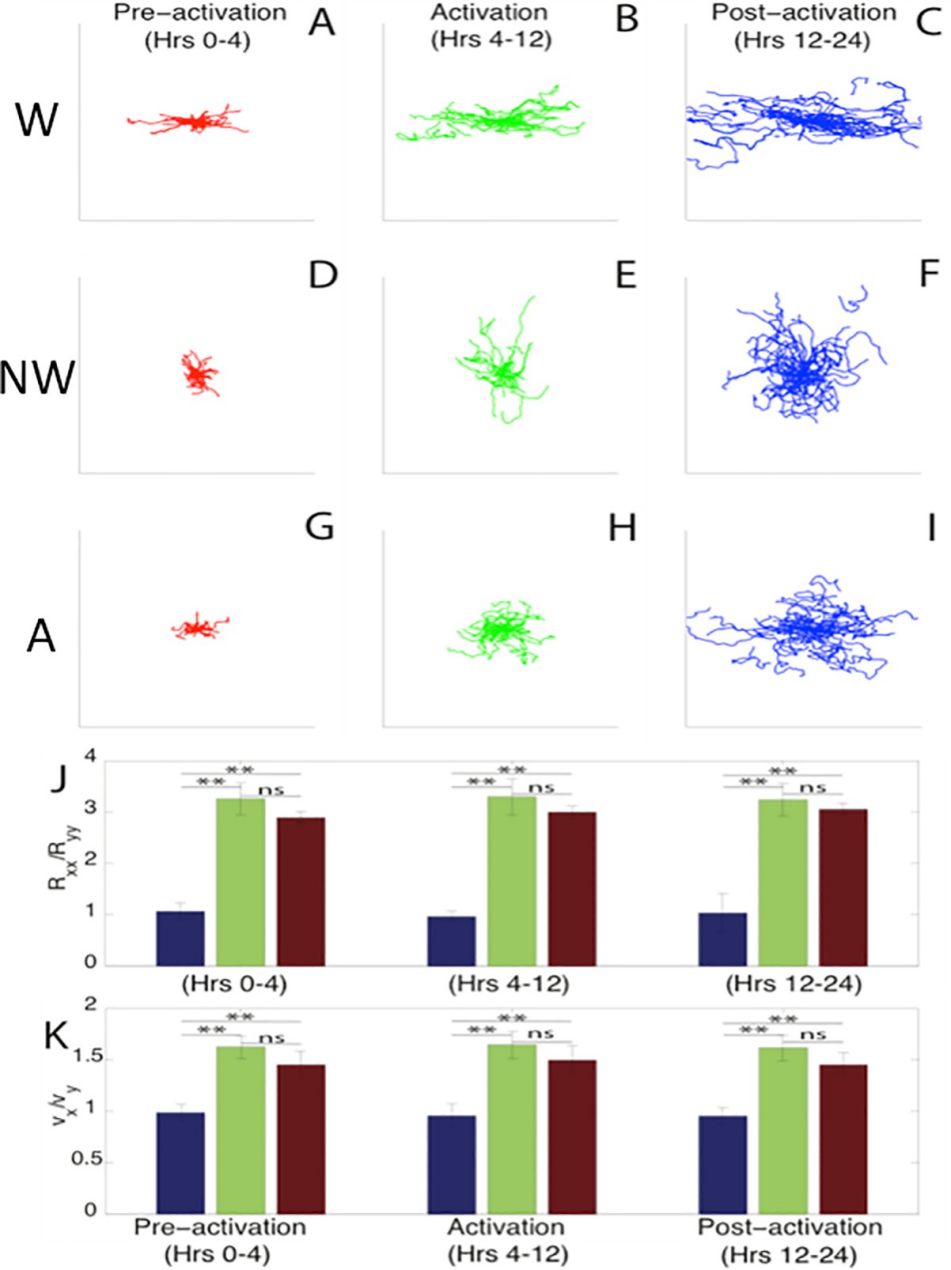
SMP trajectory statistics. (A-I) Mouse fibroblast cell trajectories with a normalized origin on wrinkled (W; A-C), non-wrinkled (NW; D-F) and active (A; G-I) substrates for each time region. Trajectories have been rotated so that the x-axis corresponds with the wrinkle direction. Cells on static non-wrinkled substrates explore space equally in all directions, while cells on static wrinkled substrates show preferred motion along the wrinkle direction. (J) Ratio of the components of the radius of gyration tensor along (R_xx) and perpendicular to (R_yy) the direction of wrinkles, quantifying the degree of anisotropy in cell trajectories on non-wrinkled (blue), wrinkled (green) and active (red) substrates. Cells on non-wrinkled substrates explore space equally in all directions, while cells on wrinkled and active substrates prefer motion along substrate wrinkles. (K) The ratio of the x and y components of mouse fibroblast velocities also quantifies the anisotropy of cell trajectories, averaged over all times. Cells on wrinkled and active substrates have significantly higher speeds along wrinkles, while cells on non-wrinkled substrates have approximately equal speeds in all directions. Single asterisks (*) indicate significance levels below 0.05, while double asterisks (**) indicate levels below 0.01. There were approximately 10^3^ cells per substrate type across 3 technical replicates and 3 biological replicates.

As we have previously observed [9], nuclear cell tracking by ACT*IV*E showed that cells on non-wrinkled substrates have no preference in how they explore space (Fig 3D–3F), while cells on both active (Fig 3G–3I) and wrinkled (Fig 3A–3C) substrates showed a strong preference to travel along the direction of forming (in the case of active wrinkles) and established (in the case of static wrinkles) wrinkle direction. We characterized cell trajectories (Fig 3) using two simple metrics which identify a directional preference. First, we calculated the radius of gyration tensor and examined the ratio between the two components along (x-direction) and perpendicular (y-direction) to the wrinkle direction for wrinkled substrates, the direction of wrinkle formation for active substrates, or a random direction for flat substrates (Fig 3J). This ratio of Rxx/Ryy corresponds to how fast cells are exploring the direction parallel to the wrinkles relative to the perpendicular direction. We see that cells on non-wrinkled substrates have a radius of gyration ratio close to 1, indicating no preference in how cells are exploring space in those environments, as expected. In contrast, both active and wrinkled substrates show values significantly higher than 1, corresponding to a strong preference to travel along the wrinkle direction. For cells on active substrates, the preference was even observed prior to thermal-activation during which time the nanotopography had not yet been fully established in the active group (Fig 2A). This indicates some measure of pattern sensing by mouse fibroblasts before transition when the SMP appears to be flat, possibly due to strain sensing or nanowrinkle formation. The ratio of cell speed along and perpendicular to wrinkles (Fig 3K), displays similar trends with regards to directional preference. Together, this indicates that cell motion is directed along the wrinkle direction.

**Fig 4:**
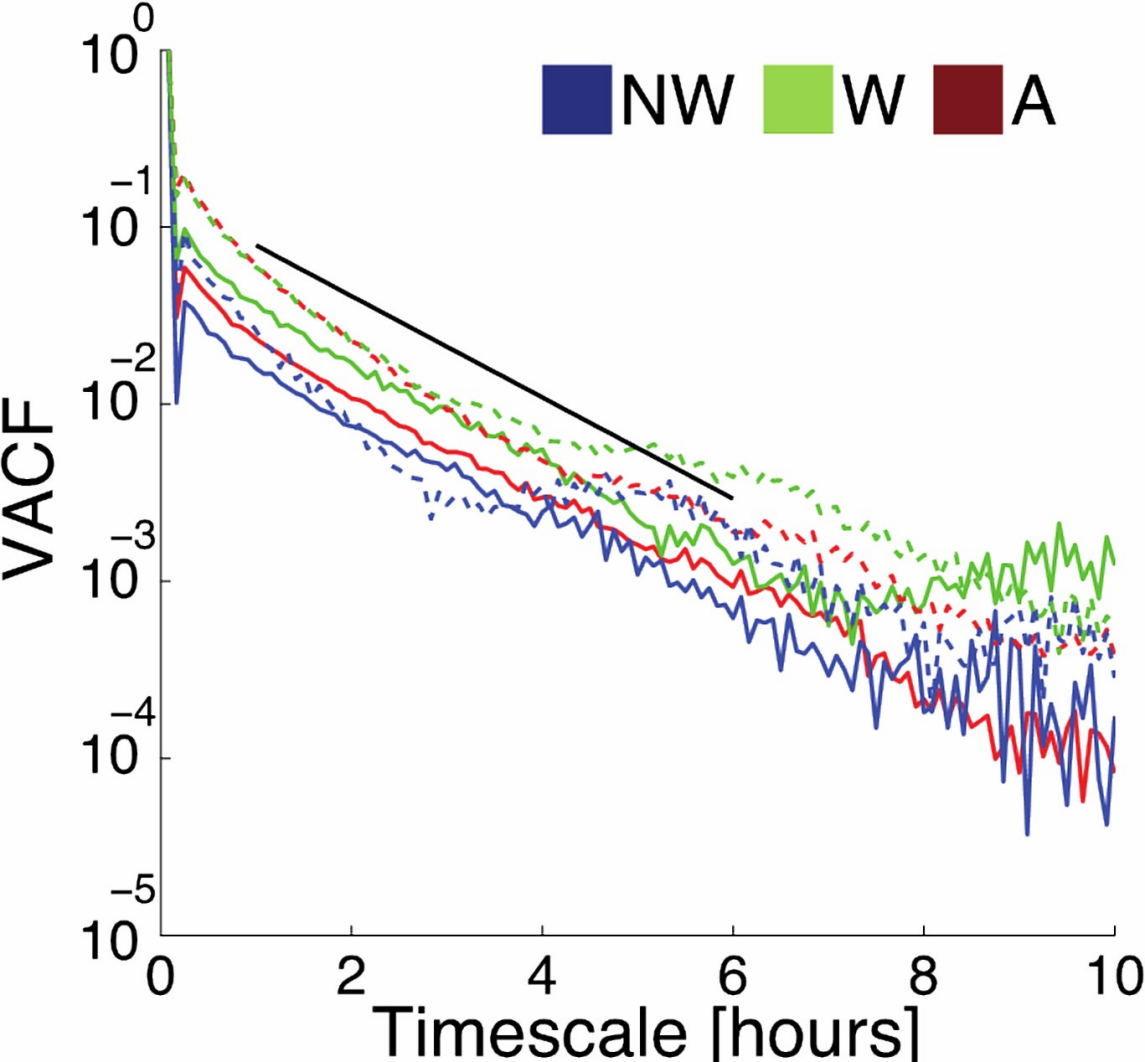
Mouse fibroblast velocity auto-correlation function. Velocity auto-correlation function (VACF) of control (solid lines) and ROCK-inhibited (dotted lines) mouse fibroblast cells on non-wrinkled (NW), wrinkled (W), and active (A) substrates. Given the proximity of each curve, we have excluded error bars in this figure so that more data would be visible. Curves are cut off at a timescale of 10 hours, after which the signal to noise ratio is low enough that results are unreliable. The black line shows a best-fit slope for all of the VACFs with an exponential decay of approximately 4 hours, corresponding to the transition timescale between ballistic and diffusive motion for trajectories in these systems. It is remarkable that even when the ROCK pathway is inhibited cells retain roughly the same diffusion timescale, indicating that ROCK-inhibition is not directly interfering with persistent cell motion, but rather the ability of cells to sense and align with wrinkles, quantified by the truncated standard deviation (TSD).

Second, we calculated the velocity auto-correlation function (VACF), which is a standard metric for quantifying the persistence time for cell trajectories (Fig 4). The slope of these lines on a semi-log plot provides a measure of the persistence time for the trajectories [30]. The persistence time was approximately 4 hours and is nearly constant across substrate type. This indicates that the presence of wrinkles does not change the inherent timescale for trajectory persistence in these fibroblasts.

### Internal cellular organization is also sensitive to active and static wrinkled substrates

**Fig 5:**
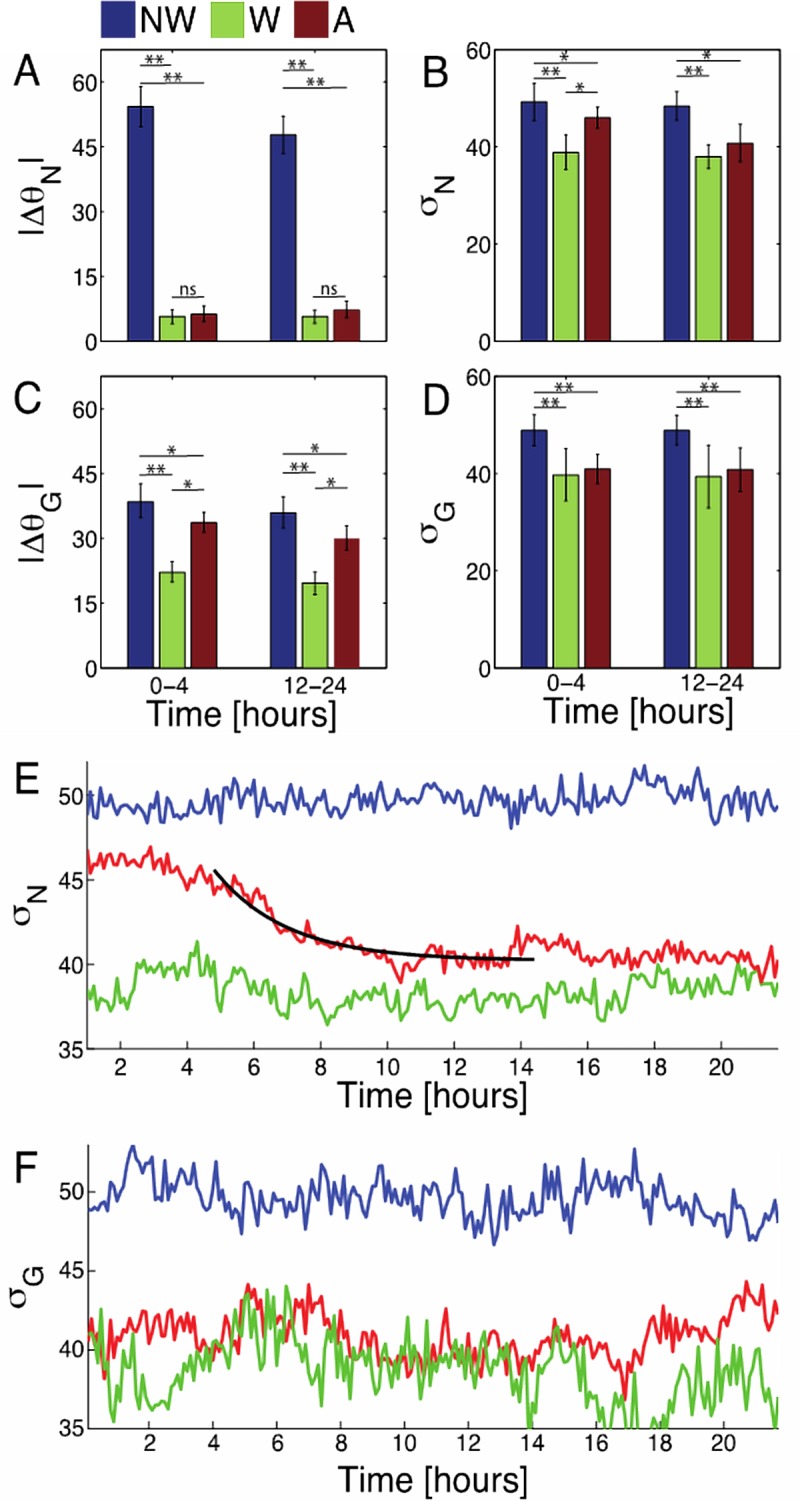
Control mouse fibroblast orientation statistics. Mean cell orientation with respect to the wrinkle direction, in degrees, (A,C) and truncated standard deviation of orientations, in degrees, (B,D) for nuclei orientation (A,B) and the axis between the nuclei and Golgi body centers-of-mass (NGV) (C,D) for control mouse fibroblast cells on non-wrinkled (NW), wrinkled (W), and active (A) substrates. Importantly, the truncated standard deviation (TSD) of nuclei orientation decreases over time, showing increasing alignment. Furthermore, the NGV has a truncated standard deviation similar to wrinkled substrates at all times, indicating that this definition of orientation could be more sensitive to environmental cues, whether pre-programmed patterns or substrate topography. (E) To elucidate the time evolution of alignment, we show a times series for nuclei truncated standard deviation, in degrees, fit with a decaying exponential (black line) with a timescale of approximately 2 hours. (F) Time evolution of NGV truncated standard deviation, in degrees. Single asterisks (*) indicate significance levels below 0.05, while double asterisks (**) indicate levels below 0.01. There were approximately 10^3^ cells per substrate type across 3 technical replicates and 3 biological replicates.

In order to better understand how cells sense and respond to nanotopographies, we measured internal markers for cell polarization and correlated them with the cell motions measured above. We first examined non-wrinkled and wrinkled static substrates (Fig 5). Using both the nuclei and NGV metrics, it is apparent that cells have a preferred direction on static wrinkled substrates (Fig 5A and 5C), with significantly closer alignment to the wrinkle direction at all times when compared to the non-wrinkled case. The TSD shows significantly higher alignment on wrinkled substrates than on non-wrinkled substrates. For example, cells on non-wrinkled substrates exhibit TSD values exceeding 50°, while TSD values in a static wrinkled system are under 40°.

We next examined the active substrates to quantify the extent to which cells respond to a changing substrate topography. We found that mouse fibroblasts on active substrates have mean nuclei orientations very close to the wrinkle direction at all times, indicating some amount of substrate-sensing during the gradual shape change that occurs before activation, confirmed by polar histograms in Fig 1. Interestingly, we still see a decrease in the active substrate nuclei TSD from values similar to a non-wrinkled substrate before activation to values similar to a wrinkled substrate after the activation. This is reinforced by the polar histograms (Fig 1A and 1B). This indicates that cells become more aligned with the wrinkle direction post-activation, as expected. The TSD for nuclei orientation exponentially decays from values statistically similar to a non-wrinkled substrate to values similar to a wrinkled substrate over a timescale of approximately 2 hours (Fig 5E). In contrast, the NGV metric shows a different trend. We see that directional alignment of the NGV, measured using TSD, is statistically equivalent between active and wrinkled substrate at all times (Fig 5F). This indicates that the NGV is already strongly aligned with the future wrinkle direction pre-activation, and, importantly, suggests that the NGV may be a more sensitive indicator of substrate features than the nuclei orientation. In fact, the behavior of the NGV mirrors the ratio of radius of gyration and velocity components (Fig 3J and 3K) respectively, both of which indicate a pre-activation alignment on active substrates.

### Inhibiting ROCK reduces the ability of cells to internally polarize on active substrates

Following our characterization of cell orientation on active substrates, we inhibit ROCK activity to determine that both nuclei shape alignment and Golgi-nuclei organization are ROCK-mediated.

We first observe that inhibiting ROCK does not change the persistence time for individual cell motion (Fig 4). The dashed lines in Fig 4 shows the ensemble-averaged VACF of ROCK inhibited trajectories, which have a similar persistence timescale (~4 hours) to that of uninhibited cells on all substrates.

**Fig 6:**
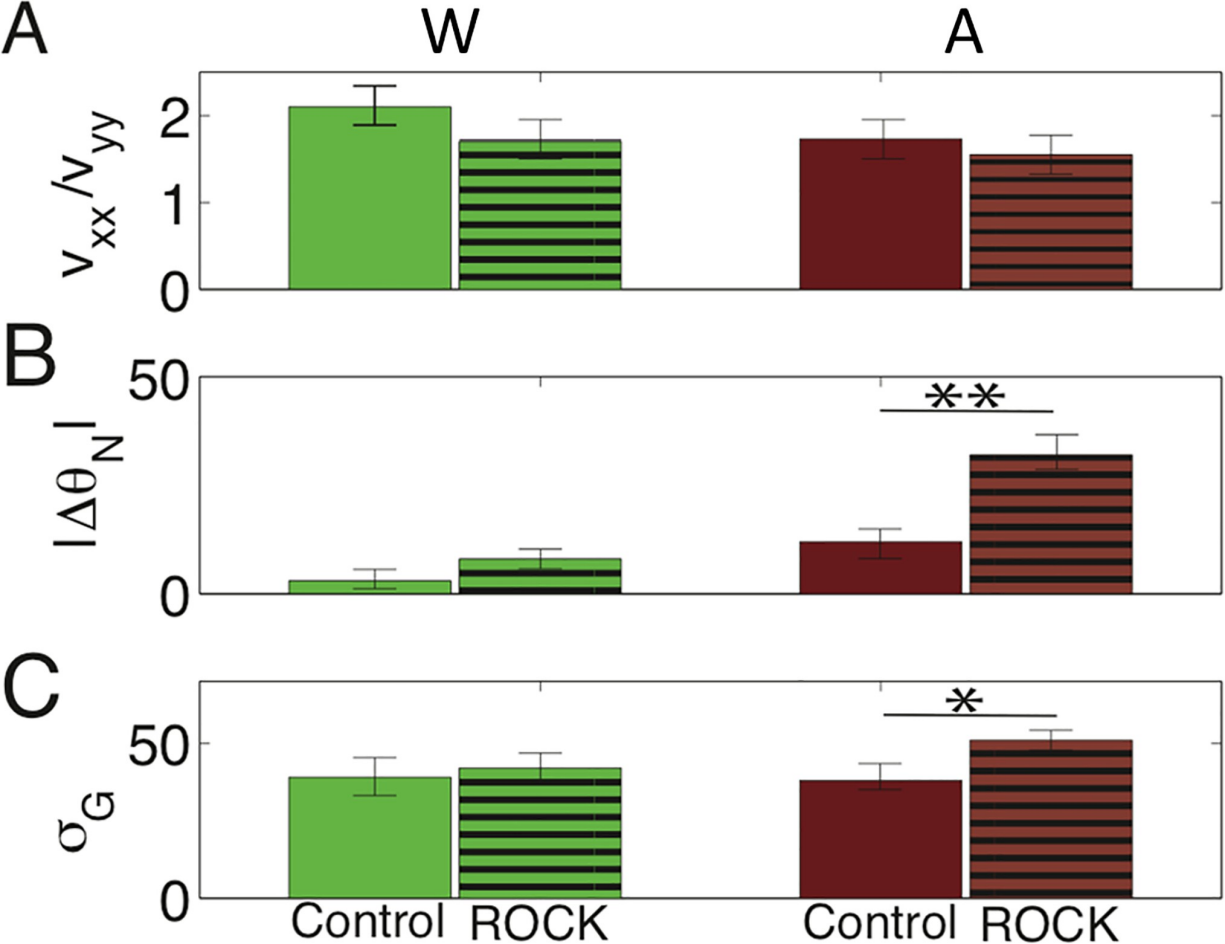
ROCK-inhibited comparisons with control mouse fibroblasts. A comparison between velocity ratio (A), mean nuclei orientation, in degrees, (B), and NGV truncated standard deviation, in degrees, for control (solid) and ROCK-inhibited (lined) systems. Inhibiting the ROCK pathway does not prevent those systems from reaching a state statistically similar to control wrinkled substrates. However, ROCK inhibition has a large effect on active substrates where it prolongs or prevents reorientation. Single asterisks (*) indicate significance levels below 0.05, while double asterisks (**) indicate levels below 0.01. There were approximately 10^3^ cells per substrate type across 3 technical replicates and 3 biological replicates.

Next, to quantify directionality, we calculated the ratio of a cell’s velocity along the wrinkle direction compared to perpendicular to that direction, on both active and wrinkled substrates, as shown in Fig 6A. We see statistically similar ratios of velocity components in all cases, suggesting that displacement of ROCK-inhibited cells is not less directed along the wrinkles.

We also compared the intracellular organization of uninhibited and ROCK-inhibited cells on wrinkled and active substrates, including the mean nuclei orientation Fig 6B, and the NGV truncated standard deviation Fig 6C. Both measures of internal structural directionality tell an interesting story: on static wrinkled substrates there is no difference between uninhibited and ROCK-inhibited cells, but ROCK-inhibited cells are significantly less directed on active substrates. In the case of mean orientation, we see a significant change in behavior for ROCK inhibited cells on an active substrate, where the mean orientation never decreases. Similarly, we see a statistically significant increase in the NGV truncated standard deviation for ROCK inhibited mouse fibroblasts. Both of these metrics point towards ROCK inhibition preventing mouse fibroblasts from sensing and responding to a patterned topography.

### Conclusions

In the present study, we quantified the relationship between nuclei orientation and the orientation of the Golgi body relative to the nucleus before, during, and after exposure to a change in SMP substrate topography designed to trigger polarized motility, and our findings suggest that the displacement vector between the nucleus and Golgi body (NGV) may be a more sensitive indicator of substrate features than the nuclei orientation. The mean orientation of the nuclei and the NGV both direct toward future or pre-existing wrinkles. Interestingly, the spread in the orientation is different between these two internal metrics: while the nuclei orientation becomes increasingly aligned during the gradual and then rapid formation of wrinkles on active substrates, the Golgi-nuclei remains aligned throughout, indicating that the NGV is more sensitive to environmental cues such as substrate strain.

These findings suggest that the two distinct metrics—nuclei orientation and the NGV—are differentially sensitive to or respond differently to substrate topography. Specifically, one possibility is that the Golgi location relative to the nucleus, and thus NGV, becomes polarized more easily than nuclei orientation, with the NGV responding to substrate topographies that do not produce a change in nuclei orientation. A second possibility is that these internal polarizations occur on different timescales. In other words, it is possible that the timescale over which the NGV responds to substrate changes is much faster than that of nuclei orientation. Distinguishing these possibilities and determining what anisotropic features of the substrate are being sensed by the cells, even before observable wrinkles form, may reveal NGV as sensitive to and a metric of strain sensing, stiffness or compliance sensing, or even sensing topographic fluctuations that are too small to be quantified with the optical microscopy used in the present work.

In this system, which was optimized to image a large number of cells simultaneously, there are fluctuations in image intensity that make the frame-to-frame displacements of nuclei somewhat noisy, and this prevents us from being able to correlate the NGV and nuclei orientation with displacements on a frame-by-frame basis; we instead focused on average quantities within a given time window. Future work could focus on increased resolution of single cells to facilitate a comparison between these two orientation metrics and overall cell displacement.

Providing some insight into the mechanisms involved, our data implicates the Rho signaling pathway, as inhibiting ROCK prevents cells from dynamically responding to topographic features on active substrates. ROCK-inhibited cells retain roughly the same truncated standard deviation at all times (S1 Fig). Coupled with comparisons to cell orientations on active substrates (Fig 6B and 6C), our study indicates that ROCK-inhibited cells are unable to sense or respond to environmental cues regardless of timescale or substrate type. However, the VACF of cell trajectories shows that ROCK-inhibited mouse fibroblast cells remain motile and retain similar persistence to uninhibited cells. In fact, it has been shown that ROCK inhibition promotes cell-cell adhesion and expansion of a wound edge in human corneal endothelial cells [31]. Similarly, our results for mouse fibroblast cells show that ROCK inhibition, while inhibiting cell-substrate interactions, does not contribute to an overall loss of motility.

The ACT*IV*E algorithm has broad applicability in organelle and cell analyses. The ACT*IV*E system has previously been employed in analyzing single and collective cell (mouse fibroblasts and HT1080) motility behaviors on static, two-dimensional patterned surfaces [9] and in dynamic, three-dimensional, shape-changing scaffolds [21]. The segmentation portion of the code has additionally been modified to investigate how shape memory dynamics positively or negatively affect cell infiltration in three-dimensional, shape-changing scaffolds [32]. The ACT*IV*E system is not limited to mammalian cell analysis; it has been adapted in its full form to track *E*. *Coli* orientation patterns [23] and motility dynamics [24] for biofilm formation applications on surfaces with varying patterns and stiffness, respectively. The current work represents a natural progression of the ACT*IV*E system, allowing for novel dual-organelle tracking that will enable insight into how intracellular dynamics directly impact single or collective cell responses.

The importance of cell motility in development, homeostasis, disease, and healing has been appreciated for some time, but with the advent of tissue engineering, regenerative medicine, and other cell-based therapies there is increased need not only to understand but also to precisely control cell motility. Here we have presented an automated image analysis technique for tracking the shape and motion of Golgi bodies and cell nuclei and to link the Golgi bodies positionally with their respective cell nuclei. We applied the technique to the study of mouse fibroblast polarization in response to changes in substrate topography, but it is important to note that the utility of the technique is not specific to the cell type (fibroblasts), micro-environment (shape-changing SMP topography), or specific process (polarization) we have studied. Moreover, the automated image analysis technique used for tracking the shape and motion of Golgi bodies in the present work could be applied to tracking of other organelles. In addition, the technique was found to be robust and capable of analyzing even low contrast, noisy images (S2 Fig). Thus, it is anticipated that the technique could be broadly employed in the study of diverse cell types, diverse micro-environments, and any cellular process involving motion of organelles and cell nuclei.

In the present work, we found that the NGV may be a more sensitive indicator of substrate features than the nuclei orientation. Based on our findings, we suggest that this metric could be a complementary tool to understand and control motility in both the fundamental fields of development, homeostasis, disease, and healing and the more emergent and applied fields of tissue engineering, regenerative medicine, and other cell-based therapies. In particular, the metric is anticipated to be useful for researchers studying the dynamics of cell polarity in response to different micro-environments.

### Appendix: Golgi tracking software

Although some of us had previously developed a software package dedicated to tracking cell nuclei (ACT*IV*E) [9], this image analysis procedure was unable to accurately identify mouse fibroblast Golgi bodies in our images due to their irregular shape and often fragmented appearance. To that end we developed a software tool to identify and track mouse fibroblast Golgi bodies and then pair these Golgi bodies with corresponding nuclei belonging to the same cell.

The first step in this software package appropriately processes lab images to account for experimental features such as phototoxicity or photobleaching. These images are recorded in a tiff stack and imported to MATLAB for analysis. For each image in a movie, we construct a distribution of pixel intensities and perform a Gaussian fit. Our goal is to adjust the brightness and contrast of each image using the imadjust function in MATLAB in an automated fashion. To that end, we estimate the input parameters for imadjust from the Gaussian distributions of pixel intensities, using values N*σ away from the distribution mean, where σ is the standard deviation and N is a user input parameter. The user must identify a value of N_high_ and N_low_ which are used to calculate the high_out and low_out input parameters for imadjust, respectively. Alternatively default values are provided which may not result in optimal performance. The software will then process the entire tiff stack and appropriately adjust the brightness and contrast to account for experimental effects and remove background noise to provide the best input for the identification component of the package.

After the images are suitably prepared, the Golgi bodies must be correctly identified in each frame. To that end we utilize the MATLAB function clusterData [25], which takes in a list of positions and a cutoff parameter, then uses a distance based clustering algorithm to group coordinates. Due to the fragmented nature of the Golgi body, we use an overly sensitive cutoff of 0.1, determined empirically from a test set of images. A complete list of user-defined parameters and their default values is given in S1 Table. The unitless sensitivity is multiplied by the maximum distance between pixels in an image to generate a distance cutoff. This means that pixel coordinates corresponding to a single Golgi body are more likely to be split apart than accurately grouped together. Following this first pass, we utilize a novel algorithm to combine all of the fragments identified by clusterData. We first examine all of the clusters the were identified in the image, remove any clusters with less than 3 pixels, and calculate the complex hull for each individual cluster to approximate its edge. Then, our algorithm begins with the largest cluster, where size corresponds to the number of pixels included, and searches near the edge for other clusters. An input parameter for this portion of the package is a distance cutoff, which the user provides as an estimate of average distance between cells in units of pixels. If the algorithm finds a cluster within this cutoff distance it will get added to the largest cluster. The cutoff distance is based on distance between cells, because the algorithm is more accurate when constructing individual Golgi bodies than when constructing multiple Golgi bodies in close proximity. After this, the convex hull of the largest cluster must be recalculated and the program looks around that edge for other clusters. We repeat this process until there are no clusters within the cutoff distance, at which point this largest cluster is taken out of the pool of candidate clusters and added to a master identification list. We return to the image, identify the largest remaining cluster and repeat the above procedure until all cluster fragments are correctly combined. To test validity, we used a sample data set of 5 frames randomly selected from each video, with approximately 15–30 Golgi bodies per frame. The tracking algorithm agreed with user identified Golgi bodies in nearly every case, with an average of 0–2 errors in each frame. The most difficult scenario is when two separate Golgi bodies from different cells are nearly on top of each other, making it almost impossible to distinguish even by eye, in which case the tracking algorithm would incorrectly group the two Golgi bodies together. Since the transfection efficiency in our system was approximately 40%, this was not a common occurrence.

Following the correct identification of Golgi bodies in individual frames across the course of a time series, we use the same Kilfoil tracking algorithm as the ACT*IV*E software package to link Golgi bodies correctly across frames, again using a distance-based algorithm where positions corresponded to Golgi body centers-of-mass.

After this was complete, we had an identification list for all of the Golgi bodies in each frame and all of the nuclei in each frame. The next portion of the software package looks at each cell nucleus and calculates the distance from it to all Golgi bodies in each frame. The user then inputs a new cutoff distance in pixels to exclude Golgi bodies which are too far away, and look at which Golgi body is, on average, the best candidate for being paired with the current nucleus. We perform this distance-based analysis for all nuclei and compare between them to make sure that there is no overlap in nuclei-Golgi body pairings and also to ensure the best pairings possible. For example, we have nucleus A which is, on average, 10 microns away from its closest Golgi body A throughout the course of the video. However, nucleus B is only 5 microns away from Golgi body A on average throughout the course of the video (these numbers are normalized by the number of frames the Golgi body is visible). In that case, the code checks and sees that the appropriate pairing is nucleus B and Golgi body A, and nucleus A defers to its second best choice assuming that there are no additional conflicts where other nuclei are closer still. This technique was quite successful in appropriately pairing nuclei and Golgi bodies to generate the nuclei Golgi vector (NGV) which is referenced in the manuscript.

## Supporting information

S1 Fig Rock-inhibited orientation statistics. Mean cell orientation (A,C) and truncated standard deviation (B,D) for nuclei orientation (A,B) and the axis between the nuclei and Golgi body centers-of-mass (C,D) for control mouse fibroblast cells on non-wrinkled (NW), wrinkled (W), and active (A) substrates. (E) Times series for nuclei truncated standard deviation (TSD), showing a similarity between cells on active and non-wrinkled substrates and an increase in TSD over time for cells on wrinkled substrates. Single asterisks (*) indicate significance levels below 0.05, while double asterisks (**) indicate levels below 0.01. There were approximately 10^3^ cells per substrate type across 3 technical replicates and 3 biological replicates.
(TIF)

S2 Fig Representative live-cell images demonstrate the robustness of the Golgi tracking technique. In the present work, the nuclear and Golgi live-cell staining was deliberately captured at low signal intensity to reduce phototoxicity and enable extended imaging to 24 h. A representative example of the nuclear (left), Golgi (middle), and RGB false colored (right) images illustrate the resulting low contrast, noisy images, which were successfully processed by the Golgi tracking code, thereby demonstrating the robustness of the approach and the potential for broad application in the study of diverse cell types, diverse micro-environments, and any cellular process involving motion of organelles and cell nuclei.
(TIF)

S1 Table User-defined input parameters for the Golgi tracking code. (PDF)
