# Supplementary figures and images for: Nuclear position relative to the Golgi body and nuclear orientation are differentially responsive indicators of cell polarized motility
Source: PLoS One. 2019 Feb 13;14(2):e0211408. doi: 10.1371/journal.pone.0211408 (PMC6373915; doi:10.1371/journal.pone.0211408)

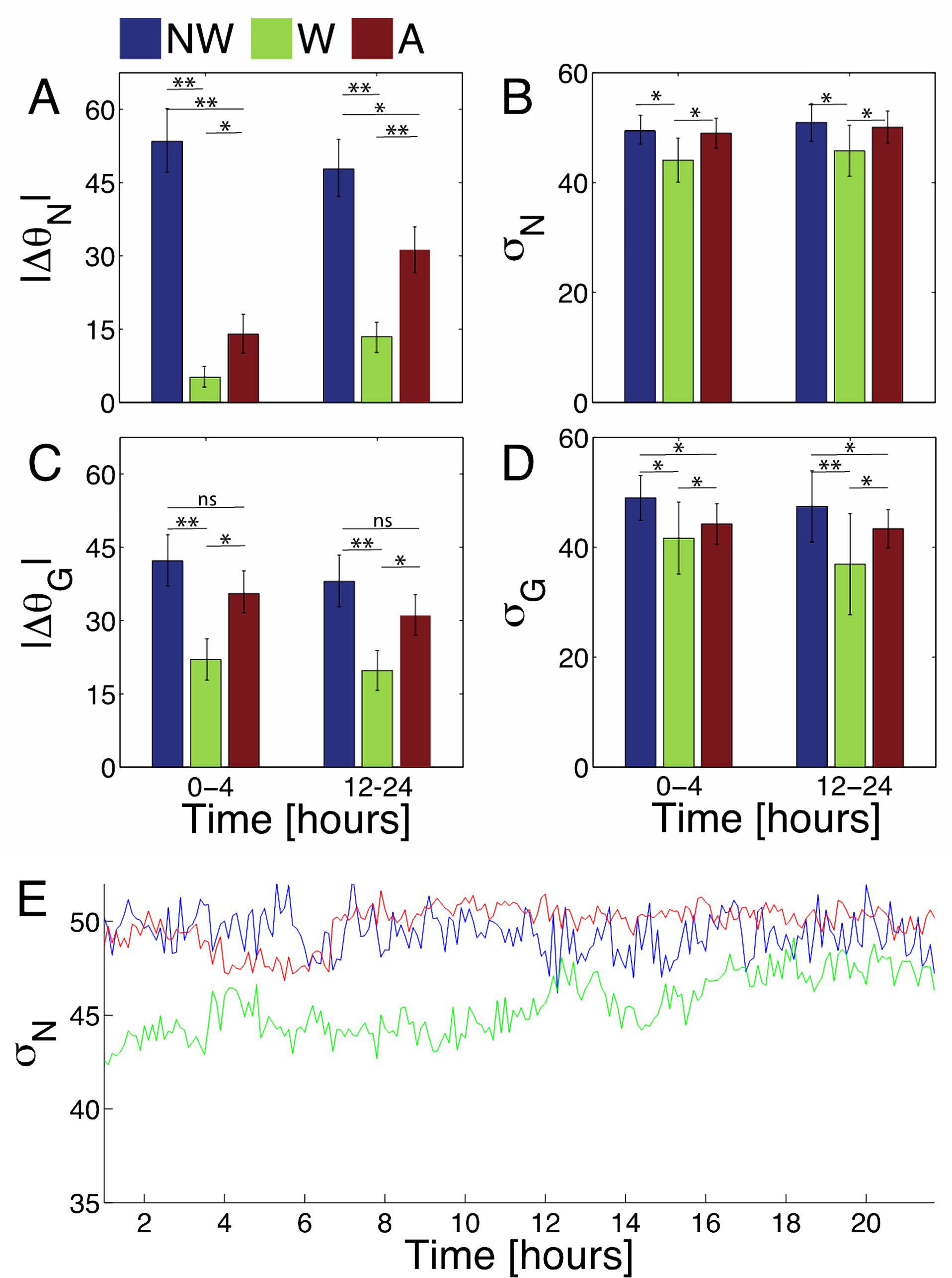

Supplement: S1 Fig — Mean cell orientation (A,C) and truncated standard deviation (B,D) for nuclei orientation (A,B) and the axis between the nuclei and Golgi body centers-of-mass (C,D) for control mouse fibroblast cells on non-wrinkled (NW), wrinkled (W), and active (A) substrates. (E) Times series for nuclei truncated standard deviation (TSD), showing a similarity between cells on active and non-wrinkled substrates and an increase in TSD over time for cells on wrinkled substrates. Single asterisks (*) indicate significance levels below 0.05, while double asterisks (**) indicate levels below 0.01. There were approximately 103 cells per substrate type across 3 technical replicates and 3 biological replicates. (TIF) [file pone.0211408.s001.tif]

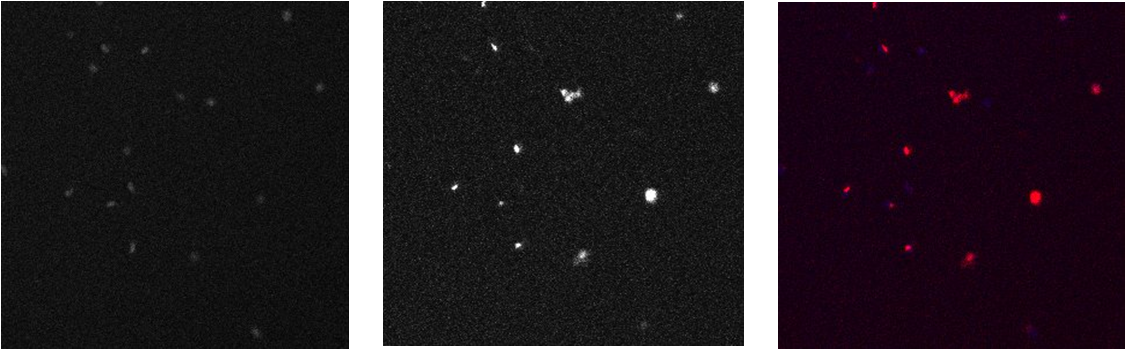

Supplement: S2 Fig — In the present work, the nuclear and Golgi live-cell staining was deliberately captured at low signal intensity to reduce phototoxicity and enable extended imaging to 24 h. A representative example of the nuclear (left), Golgi (middle), and RGB false colored (right) images illustrate the resulting low contrast, noisy images, which were successfully processed by the Golgi tracking code, thereby demonstrating the robustness of the approach and the potential for broad application in the study of diverse cell types, diverse micro-environments, and any cellular process involving motion of organelles and cell nuclei. (TIF) [file pone.0211408.s002.tif]
