# Supplementary material for: Nuclear position relative to the Golgi body and nuclear orientation are differentially responsive indicators of cell polarized motility
Source: PLoS One. 2019 Feb 13;14(2):e0211408. doi: 10.1371/journal.pone.0211408 (PMC6373915; doi:10.1371/journal.pone.0211408)
Supplement: S1 Table — (PDF) [file pone.0211408.s003.pdf]

Parameter name	Description	Units	Typical value
avg_sep	Mean separation between Golgi body fragments.	pixels	10 pixels
min_area	Cutoff area for a Golgi body fragment to be added to a larger fragment.	pixels^2	200 pixels^2
maxdisp	Maximum displacement the Kilfoil linking algorithm will search for particles between frames.	pixels	20 pixels<br>Caution: increasing this may cause errors due to combinatorics.
maxD	Maximum distance for a Golgi body and nucleus to be paired.	pixels	15 pixels
